# Personality Profiles Are Associated with Functional Brain Networks Related to Cognition and Emotion

**DOI:** 10.1038/s41598-018-32248-x

**Published:** 2018-09-17

**Authors:** Peter Mulders, Alberto Llera, Indira Tendolkar, Philip van Eijndhoven, Christian Beckmann

**Affiliations:** 1 Department of Psychiatry, Radboud University Medical Center, 6500 HB Nijmegen, The Netherlands; 2 Donders Institute for Brain, Cognition and Behavior, Centre for Cognitive Neuroimaging, 6500 GL Nijmegen, The Netherlands; 3 Radboud University Nijmegen, Comeniuslaan 4, 6525 HP Nijmegen, The Netherlands; 4 Department of Psychiatry and Psychotherapy, University Hospital Essen, 45147 Essen, Germany; 5 Oxford Centre for Functional Magnetic Resonance Imaging of the Brain (FMRIB), University of Oxford, Oxford, OX3 9DU United Kingdom

## Abstract

Personality factors as defined by the “five-factor model” are some of the most investigated characteristics that underlie various types of complex behavior. These are, however, often investigated as isolated traits that are conceptually independent, yet empirically are typically strongly related to each other. We apply Independent Component Analysis to these personality factors as measured by the NEO-FFI in 471 healthy subjects from the Human Connectome Project to investigate independent personality profiles that incorporate all five original factors. Subsequently we examine how these profiles are related to patterns of resting-state brain activity in specific networks-of-interest related to cognition and emotion. We find that a personality profile of contrasting openness and agreeableness is associated with engagement of a subcortical-medial prefrontal network and the dorsolateral prefrontal cortex. Likewise, a profile of contrasting extraversion and conscientiousness is associated with activity in the precuneus. This study shows a novel approach to investigating personality and how it is related to patterns of activity in the resting brain.

## Introduction

For the past decade, significant progress has been made in using functional neuroimaging to identify the neural correlates of human behavior. There has been a gradual shift from linking complex behavioral traits to single individual brain regions, to more recent attempts to understand behavior as complex interactions both within and between distributed sets of regions and ensuing large-scale brain networks^1^. Along these lines, advances in the use of resting-state functional magnetic resonance imaging (rs-fMRI) and initiatives to collect large openly available datasets have provided the means to investigate brain patterns without strong prior assumptions. Functional imaging during rest has the benefit of being unconstrained by specific task-based paradigms, allowing for more broad comparisons of results and more convenient data-sharing between sites^2^. Large-scale data initiatives additionally offer the increased statistical power to detect even moderate effects and to replicate results in an independent sample. Most critically, these advances together permit investigations into the neural correlates of individual traits that underlie various types of behavior.

One of the most compelling traits that influence behavior is personality, which refers to characteristic patterns of thinking, feeling and behaving. Personality is often investigated using a set of personality factors based on the “five-factor model” that includes neuroticism, extraversion, openness/intellect, agreeableness and conscientiousness^3^. These factors are empirically defined, stable over time and together integrate different psychological mechanisms that have proven useful to explain specific types of behavior^4,5^. Previous research has related these factors to structural and functional brain networks and specific related regions such as the amygdala, hippocampus and orbitofrontal cortex^6–13^. The patterns of functional interaction between these networks and regions have also been consistently identified as crucial in processes related to emotion and cognition, even during rest^14–16^. Of note, the personality factors themselves are broad summarizations of distinct underlying traits^17,18^. While these traits can be considered conceptually independent, these personality factors are not statistically independent of one another^19,20^ and empirically exhibit significant correlations. Nevertheless, research usually treats them as unitary concepts and often only reports on one or a subset of the five personality factors. This complicates interpretation when a certain personality factor might result in behavior only in a particular context, for instance in the absence of another “opposing” trait.

We can overcome this problem by considering the five personality factors as observations driven by underlying and unobserved independent cognitive and emotional processes. By applying Independent Component Analysis (ICA) in a large sample of healthy subjects from the Human Connectome Project^21^, we can identify characteristic patterns of covariation between the personality traits of the “five-factor-model”. In doing so we obtain independent personality profiles that take into account all five original traits, and subject-specific loadings for each of the new profiles. We hypothesize that these profiles can be linked to the degree of co-activation within and across brain regions associated with emotion and cognition as measured by means of resting-state fMRI.

## Results

### Personality Profiles

**Figure 1:**
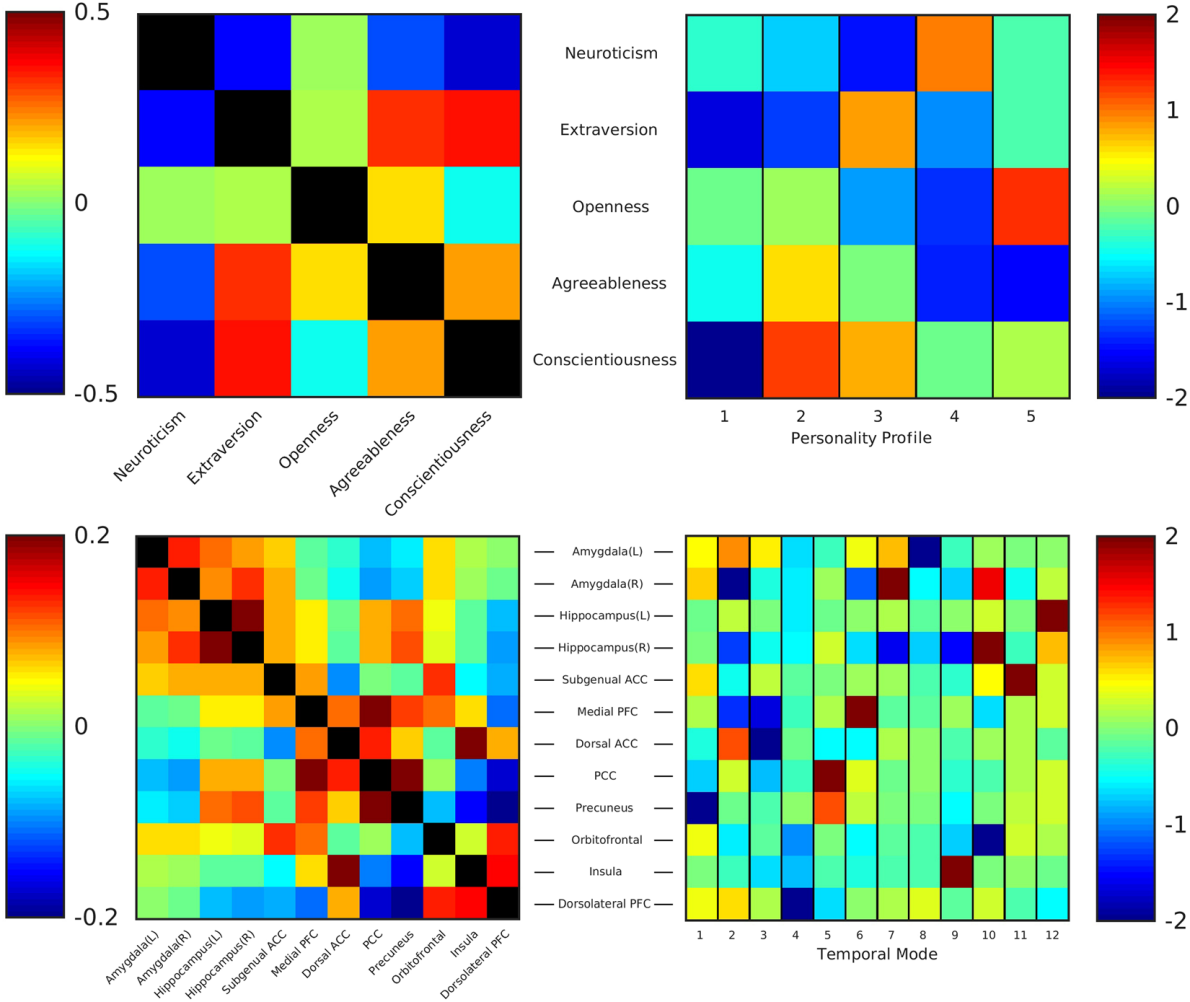
Personality and resting-state correlations and independent components. Top-left: full correlation matrix for the original personality factors. Top-right: normalized representation of the ICA-decomposition for the personality factors into new personality profiles. Each column shows an independent component with the loading onto the original personality factors. Bottom-left: full correlation matrix for the resting-state time series of the 12 regions-of-interest. Bottom-right: normalized representation of the ICA-decomposition for the resting-state time series. Each column shows an independent spatial pattern (or ‘temporal mode’) with loading onto the 12 regions-of-interest. Abbreviations: sgACC – subgenual anterior cingulate, mPFC – medial prefrontal cortex, dACC – dorsal anterior cingulate cortex, PCC – posterior cingulate cortex, PCu – precuneus, OFC – orbitofrontal cortex, dlPFC – dorsolateral prefrontal cortex.

We investigated the personality factors in 471 healthy subjects from the Human Connectome Project (age range 22–36 years) by the NEO Five-Factor Inventory (NEO-FFI) that contains 60 questions related to five different personality domains: neuroticism, extraversion, openness/intellect, agreeableness and conscientiousness. Scores for these domains and demographics are presented in Table 1. As expected, there were strong and significant correlations between several of the domains: neuroticism was negatively correlated to agreeableness, extraversion and conscientiousness; agreeableness was positively correlated to openness, extraversion and conscientiousness; extraversion was positively correlated to conscientiousness (Fig. 1).

**Table 1:** Demographics and personality factors. Personality traits and demographics of the included subjects.

Number of subjects	471
Male/Female	194/277
Age in years	29.2 (±3.51)
No twins/Twins (MZ)/Twins (DZ)	237/142/92
Education (years)	14.8 (±1.87)
NEO – Neuroticism	16.4 (±7.15)
NEO – Extraversion	30.6 (±6.12)
NEO – Openness/Intellect	28.1 (±6.11)
NEO – Agreeableness	32.1 (±4.89)
NEO – Conscientiousness	34.8 (±5.73)

Independent component analysis (ICA) on the normalized NEO-FFI personality scores was used to derive five distinct and uncorrelated personality profiles from the factor profiles (Fig. 1). These “personality profiles” have maximized statistical independence and optimally explain the variance in the behavioral data. Each of the five components contains a weight and a direction for each of the five original personality factors, but load significantly onto some factors and not onto others. Some profiles also highlight contrasting personality factors while others reveal a profile where the factors are positively related. Of note, all personality factors as measured by the NEO-FFI map onto multiple personality profiles, while each of the profiles also includes significant loadings of multiple NEO-factors. The first two personality profiles are loaded onto ‘extraversion’ and ‘conscientiousness’, either in synchrony (profile 1) or contrasting (profile 2). The next two profiles both incorporate ‘neuroticism’, but one profile also loads onto contrasting ‘extraversion’ and ‘conscientiousness’ (profile 3) while the other loads onto contrasting ‘openness’ and ‘agreeableness’ (profile 4). Finally, the fifth personality profile is defined by a contrast between ‘openness’ and ‘agreeableness’. Validation of these profiles using either a leave-one out or split-half approach show that these profiles are highly reproducible within our dataset (see Supplementary Table S2).

We use a linear projection to obtain values for every subject that reflect where they scale on each of the five new personality profiles. As an example: profile 5 loads positively onto openness/intellect and negatively onto agreeableness, with low loadings onto the other three personality factors. If the projection of this profile for a subject would be strongly positive, that subject exhibits this contrast very clearly and in the same direction (high openness/low agreeableness); if the projection was strongly negative the subject exhibits the opposite pattern (low openness/high agreeableness); if the projection is close to zero, the subject does not show a clear contrast between the original openness and agreeableness scores.

### Resting State Brain Activity

**Figure 2:**
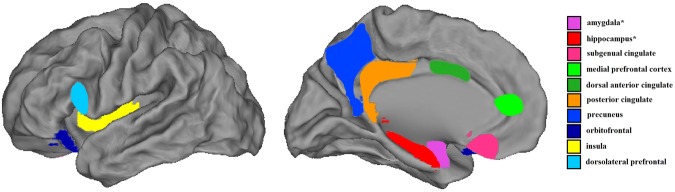
Regions-of-interest. Graphical representation of the regions-of-interest used to extract time series that are the basis the creating the temporal modes. Regions are presented on the left hemisphere. *The right amygdala and hippocampus are not represented but mirror their left equivalent.

To investigate the relationship between personality domains and brain activity, we used high-quality preprocessed resting-state data from the Human Connectome Project which includes one hour of resting-state for each subject^21^. We hypothesized that our personality profiles would be intrinsically driven by the functional interactions of networks and regions that are well established to be related to emotion and cognition on a broader scale. To test this hypothesis, we selected regions-of-interest based on their inclusion within the default mode network (medial prefrontal cortex, posterior cingulate cortex, precuneus), the salience network (insula, amygdala, dorsal anterior cingulate cortex), the cognitive executive network (dorsolateral prefrontal cortex) or on their strong relation with these networks (hippocampus, subgenual anterior cingulate cortex, orbitofrontal cortex)^15,22–24^ (Fig. 2). A multiple regions-of-interest approach, as opposed to a whole-brain analysis, increases our sensitivity to detect effects relating to our hypothesis while also decreasing the likelihood of type 1 errors. Using the Harvard-Oxford Atlas as implemented in the FSLview package, part of the FMRIB Software Library (FSL^25^, http://www.fmrib.ox.ac.uk/fsl/), we extracted the mean time series for the selected twelve regions-of-interest. Figure 1 shows the full correlation matrix for the time series of these regions and confirms the expected strong correlations between the subcortical regions, between regions within the default mode network (medial PFC, posterior cingulate cortex, precuneus) and the well-established anti-correlation between dorsolateral prefrontal cortex and default mode regions^26^. The full time series of these selected regions from all subjects were used as the basis for a temporal ICA decomposition^27^. This results in a set of characteristic patterns of activation (or “temporal modes”) that, similar to the ICA on the personality factors, have a weight and direction for each of the twelve regions-of-interest (Fig. 1). The resulting co-activation patterns reveal that some regions, such as the dorsolateral prefrontal cortex and insula, show relatively little temporal correlation with the other regions. By comparison, the right amygdala and hippocampus are incorporated into multiple temporal modes but drive none of them exclusively. Similar to the personality profiles, a leave-one and split-half validation showed that the temporal modes were highly reproducible (see Supplementary Table S2).

The temporal modes were used to obtain subject-specific “mode time series” reflecting the engagement of each patterns of co-activation at every time point. For each subject, the variance over these “mode time series” was calculated as a measure of average engagement for a particular pattern. As an example, the variance over a subjects’ projection of temporal mode 1, which is predominantly defined by activity in the precuneus, reflects a high level of engagement of the precuneus for that subject over the full hour of resting-state data. Similarly, the variance of a subject’s representation of temporal mode 3 would reflect the average engagement of a functional network defined by synchronous activity within the medial prefrontal cortex and dorsal anterior cingulate cortex.

We applied a standard linear model for each of the five personality profiles by regressing the engagement of the 12 different temporal modes to discover how our personality profiles are associated with the engagement of temporal modes of brain activity within the selected regions, while correcting for gender. Significance was assessed by permutation testing while considering the family structure, again while correcting for gender^28^. We applied false discovery rate^29^ correction to account for multiple comparisons. This analysis resulted in three personality-brain interactions. We observed that the profile defined by contrasting openness/agreeableness (profile 5) was associated with a temporal mode defined by the amygdalae, right hippocampus, medial PFC and dorsal ACC (mode 2; r = 0.16; corrected *p* = 0.015). Additionally, this same personality profile was also associated with the temporal mode that is primarily driven by the dorsolateral PFC (mode 4; r = 0.16; corrected *p* = 0.024). Furthermore, the profile relating to contrasting extraversion/conscientiousness (personality profile 2) correlated with the temporal mode defined by activity in the precuneus (mode 1; r = −0.15; corrected *p* = 0.015).

To validate the generalization of these relationships we also performed a leave one subject out approach, where we learn the group-level temporal modes and personality profiles using the data from all other subjects, and then project the left-out sample on both of these measures independently. Correlation analyses between these projected values reproduce the above described interactions (see Supplementary Table S3). In addition, the out of sample analyses reveals an additional significant relationship, namely between profile 5 and temporal mode 2.

## Discussion

It is generally appreciated that complex behaviors are rarely determined by a single variable or personality factor. Instead they result from a balance of different and sometimes opposing factors within a specific context^30^. With this in mind, investigating profiles of personality instead of personality factors in isolation might capture more distinct behavioral phenotypes and opens up many possibilities for future research. On the behavioral data alone our ICA-decomposition of personality factors as defined by the “five-factor model” gives some interesting insights into such distinct profiles of personality. The personality profiles generated by our analysis reveal how each original personality factor can be observed in the context of the other factors, which might relate to divergent types of behavior. With regards to personality, this is especially significant in cases where a clinically relevant personality factor like neuroticism results in pathological behavior (e.g. depression) specifically in the absence of a stress-resilience trait such as high conscientiousness or extraversion^31^. Of note, within our personality profiles the personality factor neuroticism is represented both in a pattern with contrasting openness and agreeableness (profile 4) and in a pattern with contrasting extraversion and conscientiousness (profile 3). The latter might be especially significant as high levels of neuroticism increases stress-sensitivity and the incidence of stress-related disorders, while extraversion and conscientiousness increase resilience to stress^31^.

We further investigate how these personality profiles relate to patterns of co-activation within and between key regions related to cognition and emotion. We show that the personality profile defined by contrasting ‘openness/intellect’ and ‘agreeableness’ is associated with two distinct patterns of co-activation in the resting brain: a temporal mode defined by the dorsolateral prefrontal cortex, key node of the cognitive executive network^23,32^ and a temporal mode resembling a self-referential/affective network defined by the amygdala, hippocampus, dorsal anterior cingulate and medial prefrontal cortex^23,33^. Regarding the personality profile, ‘openness/intellect’ is the personality factor that reflects creativity and intellectual curiosity and is related to cognitive ability and flexibility^34^. ‘Agreeableness’ includes items like compassion and politeness, and is related to positive affect and subjective wellbeing^35,36^, while low levels of agreeableness are regarded as indicative of hostility and antagonism^37^. When taken together, the personality profile we observe reflects high openness/intellect and low agreeableness on one end, and low openness/intellect and high agreeableness on the other end. Within this profile, we observe that high openness/intellect and low agreeableness relates to high engagement of the dorsolateral prefrontal cortex, which is the primary region for demanding cognitive tasks and relates to cognitive ability across a great number of experimental paradigms^38,39^. Additionally, it was also related to a temporal mode defined by interaction between the amygdala, hippocampus, medial prefrontal cortex and dorsal anterior cingulate cortex. The amygdala is well established as the key node relating to various emotional processes and has strong functional and structural connections to the adjacent hippocampus. It is also a key component of the ‘salience network’ together with the dorsal anterior cingulate cortex, which has been shown to be heavily involved in emotional processing and works together with the medial prefrontal cortex in emotion regulation^15,40^. In short, this temporal mode includes multiple regions related to emotional and self-referential processing.

We also observe an association of the personality mode defined by contrasting extraversion and conscientiousness by a temporal mode driven by activity in the precuneus, a central hub within the posterior default mode network^41,42^. ‘Extraversion’ is essentially a measure of assertiveness and social skills, while ‘conscientiousness’ is related to orderliness and industriousness. Together, this profile of personality could be interpreted as scaling from sociable or ‘easy-going’ but disorganized, to more introverted with a strong sense of duty and self-discipline. The precuneus has been implicated in a variety of functions such as consciousness, memory retrieval and self-processing^43^. It is also an important region within the default mode network and has been shown to adapt to changes in cognitive demand^41^. Overall, its function is highly adaptive and as such it has been hypothesized to orchestrate brain function at a broad level. Within our model, high levels of orderliness and low extraversion relate to strong engagement of the precuneus during rest.

Although the observed association of personality patterns with temporal modes of brain activity is highly significant, the effect sizes measured are relatively small. While this is not uncommon in neuroimaging, it underlines that while mathematical models might help us understand behavior better, we are limited by our ability to quantify behavior and by how we measure activity in the brain. We are also limited by our focus on regions-of-interest as we cannot exclude the possibility that other regions might contribute to personality or the observed modes of resting-state activity. Note here that we consider “engagement” as represented by the variance over the temporal mode series. Consequently, the presented model is a simplification and does not account for stable “low” or “high” states over the full hour of rest. Those dynamics could be better understood by employing different mathematical models to take into account the time structure (i.e. Hidden-Markov Models), and is outside of the scope of this paper. Finally, although we show that our findings are reproducible within our dataset, applying a similar model to an independent dataset is needed to validate our results.

In summary, we investigate personality in a large healthy cohort and show that it is possible to capture distinct profiles of personality. These profiles have the benefit of observing the original personality factors in the context of all other traits, which could prove useful in linking personality to, for example, increased risk for psychiatric disorders. We also show that two of these ‘personality profiles’ are associated with patterns of co-activation in the brain during rest in regions that are known to be involved with cognition and emotion. These findings provide new ways for investigating patterns in behavior and how they relate to brain function which can lead to a better understanding of both normal and pathological processes.

## Methods

### Study population

In this study, we used data from the Human Connectome Project (HCP) 500-subjects release^21^. The HCP project (Principal Investigators: Bruce Rosen, M.D., Ph.D., Martinos Center at Massachusetts General Hospital; Arthur W. Toga, Ph.D., University of Southern California, Van J. Weeden, MD, Martinos Center at Massachusetts General Hospital) is supported by the National Institute of Dental and Craniofacial Research (NIDCR), the National Institute of Mental Health (NIMH) and the National Institute of Neurological Disorders and Stroke (NINDS). HCP is the result of efforts of co-investigators from the University of Southern California, Martinos Center for Biomedical Imaging at Massachusetts General Hospital (MGH), Washington University, and the University of Minnesota. The HCP includes data from healthy adult twins and their non-twin siblings. We included all data from subjects that had resting-state fMRI available for all four time-points and completed the NEO-FFI personality inventory. Two subjects were excluded on the basis of structural abnormalities. The considered data was gathered from 194 males and 277 females with a small age range (range 22–36 y, mean 29.2 y).

### Personality scores

The NEO-FFI contains 60 questions divided into 12 questions relating to 5 different personality domains: ‘neuroticism’, ‘extraversion/introversion’, ‘openness to experience’, ‘agreeableness’ and ‘conscientiousness’^44,45^. The NEO-FFI has shown excellent reliability and validity^4^. The data used in this study was collected as part of the Penn Computerized Cognitive Battery^46,47^ within the HCP.

### MRI data acquisition

We used resting-state fMRI-data from the Human Connectome Project (HCP)^21^. This contains one hour of resting-state data for each subject (4800 time points), obtained in four 15-minute block across two visits, with each visit having separate scans for both encoding directions (left-right and right-left). Scans were obtained using a customized Siemens 3 Tesla “Connectome Skyra” housed at Washington University in St. Louis, using a standard 32-channel Siemens receiver head coil. with a gradient-echo simultaneous multi-slice EPI sequence. This sequence has the following characteristics: TR 720 [ms]. TE 33.1 [ms], flip angle 52 [deg], field of view 208 × 180 [mm], voxelsize of 2.0 [mm^3^], multiband factor 8, echo spacing 0.58 [ms]. For this study, we included the 471 subjects that had all 4 scans available and completed the questionnaires on personality domains (NEO-FFI).

### Personality data analysis

NEO-scores and demographics are presented in Table 1. We appreciate that there were significant Pearson correlations between several of the domains: neuroticism was negatively correlated to agreeableness, extraversion and conscientiousness; agreeableness was positively correlated to openness, extraversion and conscientiousness; extraversion was positively correlated to conscientiousness (Fig. 2). The NEO-scores for all 471 subjects were used as an input for ICA using the fastICA algorithm (at full rank)^27^, which resulted in five independent components that represent personality profiles derived from the behavioral data. These were multiplied with the original data to obtain, for each subject, a loading for each component that represents where they scale on each of the five new personality profiles. For the personality profiles, we checked if these were related to motion and found that one (personality profile 4) showed a significant correlation to the root mean-squared of the relative displacement (r = 0.15, fdr-corrected *p* = 0.0495). We found no relation of motion to the personality profiles that were significantly correlated with resting-state data.

### Resting state fMRI preprocessing

We used the minimally preprocessed resting-state fMRI data from the HCP. This includes gradient distortion correction, FLIRT-based motion correction^48^, TOPUP-based field map preprocessing^49^, distortion correction and registration of the EPI to high resolution T1-weighted image using a Boundary Based Registration algorithm^50^, spline resampling from EPI to standard space and intensity normalization and bias field removal. Additional preprocessing to remove structured noise was performed using ICA-FIX^51,52^.

### Resting state fMRI analysis

All fMRI analysis was performed using the FMRIB Software Library (FSL)^25,53^. In this work we perform spatial dimensionality reduction prior to a temporal ICA decomposition. For spatial dimensionality reduction we selected regions of interest based on prior literature and the Harvard-Oxford atlas^54^. We opted for a regions-of-interest approach to decrease the chance for type I errors and to increase our sensitivity to detecting associations between our new and complex behavioral modalities and the brain. For each region, we created a mask for the area with high (>50%) probability of belonging to that specific region. For the anterior cingulate cortex (ACC), we made a sub-selection for the dorsal ACC (dACC) and the pregenual/medial prefrontal section (mPFC). This resulted in 12 regions of interest: amygdala (left/right), hippocampus (left/right), subgenual anterior cingulate cortex (sgACC), medial prefrontal cortex (mPFC), dorsal anterior cingulate cortex (dACC), posterior cingulate cortex (PCC), precuneus (PCu), dorsolateral prefrontal cortex (dlPFC), insula and orbitofrontal cortex (OFC) (Fig. 1).

We extract the mean time-series reflecting the temporal dynamics of each of the regions of interest and perform a full rank temporal ICA decomposition^27^ to identify spatial patterns of interaction and their related independent time-series. This approach can be seen as a spatially informed Temporal Functional Modes analysis^55^. We will further refer to these time-series as ‘mode time-series’.

### Resting state fMRI data association with personality

For each subject, we calculated the variance over the twelve mode time-series and perform a linear correlation analysis between these variances and the personality dimensions as reported above; corrected for the effect of gender. We identified relations of interest at *p* < 0.05 level, after false discovery rate correction to account for multiple comparisons^29^. These relationships are then tested by means of ‘permutation analysis of linear models’ (PALM, 100,000 permutations)^28^ to take into account the family structure of the HCP, again correcting for gender effect. The full table of the five personality profiles and temporal modes are presented in Table S1.

### Validation

To evaluate the reproducibility of the reported personality profiles and temporal modes we applied both a leave-one out and split-half validation (including data normalization and ICA). We report the mean correlation and standard deviation with respect to the results obtained from the analysis on the full sample (as reported in the paper). In addition, for the split-half validation, we also present the mean and standard deviation of the correlations between the solutions obtained using each of two half samples considered. These results are presented in Supplementary Table S2.

To validate the correlations between personality profiles and temporal modes we used a leave-one-subject-out approach. For each subject, we learn the group-level temporal modes and personality profile using the data from all other subjects, and then project the left-out sample on both of these measures independently. We then correlate these projected values between the personality profiles and temporal modes. The p-values for this correlation can be found in Supplementary Table S3. The three interactions we report in the manuscript are clearly reproducible within our sample. Furthermore, we observe one significant interaction (between temporal mode 7 and profile 5) that did not survive statistical threshold before.

## Electronic supplementary material

Supplementary information

## Data Availability

All personality scores and resting-state fMRI data are available at http://www.humanconnectome.org. The Harvard-Oxford atlas used to define ROIs is available as part of the FSL toolbox at http://fsl.fmrib.ox.ac.uk/fsl/fslwiki/Atlases. Both the FastICA and PALM algorithms are available online^27,28^. Any additional information or resource is available on request.
