# Supplementary material for: Personality Profiles Are Associated with Functional Brain Networks Related to Cognition and Emotion
Source: Sci Rep. 2018 Sep 17;8:13874. doi: 10.1038/s41598-018-32248-x (PMC6141550; doi:10.1038/s41598-018-32248-x)
Supplement: Supplementary file 1 — Supplementary information [file 41598_2018_32248_MOESM1_ESM.pdf]

Supplemental information for:

Personality Profiles Are Associated with Functional Brain Networks Related to Cognition and Emotion

Peter Mulders[1,2], Alberto Llera[2], Indira Tendolkar[1,2,3], Philip van Eijndhoven[1,2,4], Christian F. Beckmann[2,4]

[1] Department of Psychiatry, Radboud University Medical Center, 6500 HB Nijmegen, the Netherlands

[2] Donders Institute for Brain, Cognition and Behavior, Centre for Neuroscience, 6500 GL Nijmegen, the Netherlands

[3] Department of Psychiatry and Psychotherapy, University Hospital Essen, 45147 Essen, Germany

[4] Shared last authorship

Correspondence

petercr.mulders@radboudumc.nl

	TM1	TM2	TM3	TM4	TM5	TM6	TM7	TM8	TM9	TM10	TM11	TM12
**Profile 1 'ext & con'**	0.54	0.27	0.28	0.67	0.52	0.99	0.59	0.90	0.87	0.75	0.18	0.80
**Profile 2 'ext x con'**	0.0012* *0.0007***	0.52	0.45	0.73	0.41	0.020	0.16	0.36	0.64	0.90	0.12	0.89
**Profile 3 'neu x ext/con'**	0.31	0.68	0.96	0.012	0.34	0.79	0.84	0.60	0.90	0.90	0.35	0.44
**Profile 4 'neu x ope/agr'**	0.93	0.81	0.23	0.18	0.60	0.85	0.027	0.48	0.55	0.94	0.41	0.23
**Profile 5 'ope x agr'**	0.41	0.00029* *0.0096***	0.18	0.00051* *0.0026***	0.84	0.37	0.013	0.17	0.40	0.79	0.66	0.16

Table S1. Statistics output. Results (p-values) for regression analysis of the five personality dimensions versus the 12 temporal modes as presented in figure 1, corrected for gender. Abbreviations: ext: extraversion; con: conscientiousness; ope: openness/intellect; agr: agreeableness; neu: neuroticism; TM: temporal mode. * *significant interactions at $p < 0.05$ after FDR-correction. ** p-values after permutation analysis of linear models to account for family structure of the data.*

	Leave-one out		Split-half		Split-half versus full	
	mean	std	mean	std	mean	std
**Personality profile 1**	0.98	0.03	0.83	0.11	0.95	0.07
**Personality profile 2**	0.95	0.03	0.83	0.12	0.86	0.12
**Personality profile 3**	0.97	0.02	0.79	0.10	0.92	0.06
**Personality profile 4**	0.95	0.06	0.80	0.13	0.87	0.09
**Personality profile 5**	0.91	0.14	0.72	0.10	0.79	0.12
**Temporal mode 1**	0.95	0.03	0.86	0.07	0.95	0.03
**Temporal mode 2**	0.99	0.02	0.93	0.05	0.86	0.15
**Temporal mode 3**	1.00	0.01	0.87	0.09	0.93	0.07
**Temporal mode 4**	0.99	0.01	0.85	0.15	0.96	0.06
**Temporal mode 5**	1.00	0.00	0.80	0.15	0.96	0.03
**Temporal mode 6**	0.94	0.09	0.87	0.12	0.84	0.12
**Temporal mode 7**	0.94	0.03	0.83	0.12	0.92	0.05
**Temporal mode 8**	0.98	0.01	0.82	0.19	0.95	0.04
**Temporal mode 9**	0.89	0.89	0.81	0.11	0.80	0.13
**Temporal mode 10**	0.82	0.82	0.81	0.11	0.74	0.10
**Temporal mode 11**	0.98	0.01	0.73	0.13	0.97	0.01
**Temporal mode 12**	0.98	0.02	0.74	0.19	0.89	0.10

Table S2. Reproducibility of personality profiles and temporal modes. Mean correlation and standard deviation for the reproducibility of the personality profiles and temporal modes. For the leave-one out approach, correlation represents the average correlation between the full ICA results and each iteration where one subject is excluded. For the split-half validation, the middle column ("Split-half") represents the correlation between the results when ICA was performed on two halves of the dataset independently, while "split-half versus full" represents the correlation between results obtained from the ICA on half the data against the original analysis on the full sample. Highlighted rows show the profiles and modes that relate to the significant brain-behavior correlations. We show here that (1) both leave-one out and split-half reproducibility is high, for both the personality profiles and the temporal modes and (2) the level of reproducibility is dependent on the amount of data used, as is apparent by the difference in reproducibility between columns 1 and 2-3; *Abbreviations: std: standard deviation.*

	TM1	TM2	TM3	TM4	TM5	TM6	TM7	TM8	TM9	TM10	TM11	TM12
**Profile 1 'ext & con'**	0.71	0.25	0.44	0.14	0.30	0.87	0.11	0.22	0.94	0.38	0.19	0.69
**Profile 2 'ext x con'**	*0.0008**	0.15	0.48	0.52	0.47	0.049	0.090	0.18	0.67	0.96	0.070	0.61
**Profile 3 'neu x ext/con'**	0.82	0.62	0.29	0.71	0.53	0.36	0.11	0.32	0.79	0.64	0.31	0.75
**Profile 4 'neu x ope/agr'**	0.82	0.64	0.21	0.47	0.63	0.66	0.074	0.81	0.44	0.64	0.41	0.29
**Profile 5 'ope x agr'**	0.19	*<0.0001**	0.18	*<0.0001**	0.96	0.073	0.0004*	0.0044	0.43	0.010	0.55	0.016

Table S3. Results for the validation. p-values from the approach used to validate the original findings. Subjects' loading onto the personality profiles and variance over the temporal modes were predicted out of sample, and these values were correlated to validate the findings of the original analysis. Highlighted cells relate to the significant correlations from the original analysis. Abbreviations: ext: extraversion; con: conscientiousness; ope: openness/intellect; agr: agreeableness; neu: neuroticism; TM: temporal mode. * *significant interactions at $p < 0.05$ after FDR-correction.*
